# Body Recomposition Effects of Long-Term Glycyrrhizin Consumption in Nonobese Individuals: From the Clinic to the Bench

**DOI:** 10.1021/acsptsci.5c00120

**Published:** 2025-07-15

**Authors:** Yang-Ching Chen, Yu-Cih Huang, Yu-Jie Cheng, Jessika Woo Kar Man, Rong-Hong Hsieh, Shih-Yuan Hsu, Yue-Hwa Chen

**Affiliations:** a Department of Family Medicine, Wan Fang Hospital, Taipei Medical University, Taipei 116, Taiwan; b Department of Family Medicine, School of Medicine, College of Medicine, 38032 Taipei Medical University, Taipei 110, Taiwan; c School of Nutrition and Health Sciences, College of Nutrition, 38032 Taipei Medical University, Taipei 110, Taiwan; d Graduate Institute of Metabolism and Obesity Sciences, 38032 Taipei Medical University, Taipei 110, Taiwan; e Nutrition Research Center, Taipei Medical University Hospital, Taipei 110, Taiwan; f School of Food Safety, College of Nutrition, 38032 Taipei Medical University, Taipei 110, Taiwan; g Research Center of Food Safety Inspection and Function Development, College of Nutrition, 38032 Taipei Medical University, Taipei 110, Taiwan

**Keywords:** glycyrrhizin, body fat, fat-free mass, preadipocyte differentiation, muscle growth

## Abstract

Non-nutritive sweeteners are used for obesity management, but their benefits and risks are unclear. Artificial sweeteners may harm cardiovascular health, while natural sweeteners like glycyrrhizin offer potential benefits. This study examined long-term glycyrrhizin consumption’s effects on body composition in adolescents and mice, comparing obese and normal-weight individuals and exploring underlying mechanisms. Data from the Taiwan Pubertal Longitudinal Study (TPLS) (*n* = 1641) were analyzed, and experiments with C57BL/6 mice and 3T3-L1 preadipocytes were conducted. Higher glycyrrhizin consumption correlated with lower body fat and higher fat-free mass in adolescents, especially nonobese individuals. In mice, glycyrrhizin supplementation reduced adipose tissue weight and serum leptin and cholesterol levels and increased muscle weight and MyoG mRNA expression. Cell experiments showed that glycyrrhizin inhibited adipocyte differentiation and lipid accumulation in preadipocytes. The mechanism involved reduced expression of mRNAs such as C/EBPα, GLUT4, leptin, and adiponectin. Glycyrrhizin consumption may reduce adiposity and increase muscle mass in nonobese individuals by inhibiting adipocyte differentiation. These findings suggest that glycyrrhizin influences body composition by reducing fat mass and increasing muscle mass in nonobese individuals, warranting further clinical studies.

## Graphical abstract

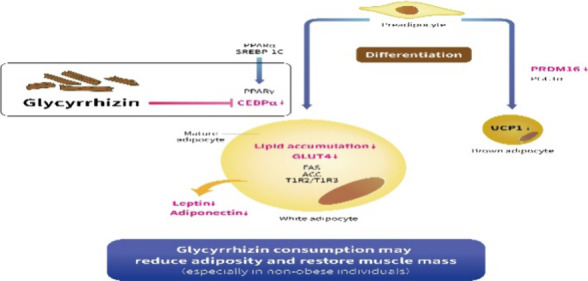

Although non-nutritive sweeteners (NNSs) have been increasingly used worldwide as an alternative to sugar to help individuals with obesity control their caloric intake, the benefits and risks of these compounds remain unclear.^1^ In a meta-analysis of cohort studies, Azad et al.^2^ reported that consumption of NNSs was associated with obesity. In another meta-analysis of randomized controlled trials, Laviada-Molina et al.^3^ discovered that overweight or obese individuals who consumed NNSs experienced a significant reduction in weight. Common artificial sweeteners may adversely affect cardiovascular health by inducing glucose intolerance^4^ and increasing the risk of cardiovascular disease.^5^ By contrast, naturally derived sweeteners such as glycyrrhizin or steviol can lower hyperglycemia and enhance lipid metabolism.^2^ To the best of our knowledge, no human studies have yet elucidated the adiposity-reducing effects of glycyrrhizin.

Adipogenesis is a multistep process where preadipocytes differentiate into mature adipocytes, involving tightly regulated transcriptional cascades. Key transcription factors such as CCAAT/enhancer-binding proteins (C/EBPβ, C/EBPδ) initiate the early phase, which then activates peroxisome proliferator-activated receptor gamma (PPARγ) and C/EBPα to drive terminal differentiation and lipid accumulation. These transcription factors regulate downstream adipogenic genes like Glucose Transporter Type 4 (GLUT4), aP2, and adiponectin, contributing to the adipocyte phenotype.^6–8^ Moreover, signaling pathways such as MEK/ERK and AMPK have been implicated in modulating adipogenic progression. MEK/ERK promotes clonal expansion and differentiation by enhancing C/EBPβ/δ expression, while AMPK activation inhibits adipogenesis by suppressing PPARγ and C/EBPα.^9^ Given the rising prevalence of obesity and the emerging interest in dietary sweeteners, exploring how natural NNSs like glycyrrhizin influence these molecular events is of particular relevance.

Glycyrrhizin, also known as glycyrrhizic acid (GL), is a primary bioactive compound in licorice roots and is widely used in confectionery and herbal medicines because of its sweetness, which is approximately 50 times that of sucrose.^10^ GL is typically used in a salt form, such as that of monoammonium glycyrrhizinate (MAG), because of its higher sweetness (50–100 folds of sucrose) of water solubility.^11^ Because it is inexpensive to extract and has high purity, it is widely used in low-calorie beverages and foods. Glycyrrhizin has various beneficial physiological functions, including antioxidative,^12^ anti-inflammatory,^13^ antiatherosclerotic,^14^ hepatoprotective,^15^ and antiadipogenic^12^ effects. According to the literature, GL can reduce body weight gain; reduce the size of adipocytes in adipose tissue; and improve blood levels of glucose, insulin, leptin, and lipids in rats subjected to high-sucrose or high-fat diets (HFDs).^16^

To our knowledge, no longitudinal human studies have yet examined the effects of glycyrrhizin on body composition. Furthermore, the majority of studies on glycyrrhizin have involved obese animal models; the effects of glycyrrhizin have not yet been compared in normal-weight and obese groups. In this study, we explored the molecular mechanisms underlying the effects of glycyrrhizin. We hypothesized that inhibition of preadipocyte differentiation and adipocyte adipogenesis would be associated with reduced lipid accumulation in adipocytes.^7,17^ Overall, our goals were to (1) determine the long-term effects of glycyrrhizin consumption on body recomposition in adolescents and mice, (2) compare these effects in obese and normal-weight groups, and (3) identify potential mechanisms underlying these effects by using models of mature adipocytes and preadipocyte differentiation.

## Methods

### Human Study

#### Study Design and Data Collection

The Taiwan Pubertal Longitudinal Study (TPLS) is a multidisciplinary longitudinal project (open cohort) that has enrolled 2413 children from puberty and pediatric endocrine clinics in Taipei (Taipei Medical University Hospital, Taipei Municipal Wanfang Hospital, and Cathay General Hospital) and Tainan (National Cheng Kung University) since 2018. This study excludes subjects with metabolic disorders and congenital conditions, such as diabetes, maple syrup urine disease, and phenylketonuria. The data herein involved 1641 children with complete data of Glycyrrhizin consumption and body composition. (Figure S1) In this study, adolescents aged 7–14 years were invited to participate and were prospectively followed up every 3 months to monitor changes in their body composition at their respective hospitals. Tanner’s stage, physical activity levels, sleep duration and quality, and anthropometric measures were recorded every 3 months during routine clinic visits. Informed consent was obtained from parents or guardians, and adolescents provided assent before participation. This study was approved by the Institutional Review Board of Taipei Medical University (N201911016), CGH (CGH-P108107), and NCKUH­(B-BR-108–076) and complied with the principles outlined in the Helsinki Declaration.

#### Exposure and Outcome Assessment

At baseline, 24-h dietary recall was conducted by trained, registered dietitians. Total energy and nutrient intake were estimated using COFIT Pro Nutritionist Edition version 1.0.0, a nutrient analysis software package that features a food composition table as a nutrient database. Additional information regarding the validity of COFIT Pro is provided in our previous study.^18^

Glycyrrhizin consumption was evaluated using the NNS Food Frequency Questionnaire.^19^ To develop a valid tool for evaluating the consumption of NNSs, an extensive market survey was conducted to create a comprehensive and semiquantitative food frequency questionnaire specifically for evaluating glycyrrhizin intake. This questionnaire includes a 39 glycyrrhizin-containing food and beverage products.^19^ The average frequency per week and the amount of NNS consumed over the past 3 months were calculated. Glycyrrhizin intake was expressed as a proportion of the acceptable daily intake (ADI) set by the Joint FAO/WHO Expert Committee on Food Additives (JECFA).^20^ JECFA set the No-Observed-Adverse-Effect Level (NOAEL) for calculating ADI of glycyrrhizin to be 2 mg/kg bw/d. The daily intake of Glycyrrhizin was divided by the body weight of the participant (kg) to yield a body weight–adjusted intake (mg/kg/day), and this value was expressed as a proportion of the ADI for glycyrrhizin (0.2 mg/kg/day, as defined by the Joint FAO/WHO Expert Committee on Food Additives). In this study, the medium level of glycyrrhizin consumption observed in the TPLS study is 0.11%ADI. Participants were divided into three groups on the basis of their glycyrrhizin consumption: no glycyrrhizin intake, glycyrrhizin intake less than or equal to the median (low consumption), and glycyrrhizin intake greater than the median (high consumption).

Body weight and composition (percentage of fat mass and fat-free mass) were measured in light clothing by using a portable bioimpedance electronic scale (TT-BC418; TANITA, Tokyo, Japan). Flexible tapes were used to measure the circumference of the waist and hips to the nearest millimeter. Body mass index (BMI, kg/m^2^) values were transformed into *z*-scores by using age- and sex-related reference charts.^21^ Cutoff points for age- and sex-specific 95th percentiles were used to define obesity following the Growth Charts for Taiwanese Children.^22^

#### Covariate Assessment

The covariates included in the statistical models were a priori confounders identified from previous research with data relevant to both glycyrrhizin consumption and body composition. All models were adjusted for sex, age, physical activity level, sleep quality, total energy intake, and parental educational level. Physical activity levels were measured using the International Physical Activity Questionnaire.^23^ Sleep quality was evaluated using the Pittsburgh Sleep Quality Index,^24^ with higher scores indicating lower sleep quality.

### Animal Experiment

#### Study Design

Fifty-six male C57BL/6 mice aged 4 weeks were purchased from BioLASCO (Taipei, Taiwan) and maintained at a temperature of 22 °C ± 2 °C and relative humidity of 55% ± 10% under a 12-h light/dark cycle with 4 mice per cage. The mice were first fed either a standard AIN-93M diet or an HFD (60% fat; MP Biomedicals, Irvine, CA, USA) based on AIN-93M for 8 weeks to maintain normal weight or induce obesity, respectively. Following this period, mice on the standard diet received drinking water containing 0, 1.1, and 3.3 g/L of MAG (Zhangjiagang Free Trade Zone Mafco Biotech, Jiangsu, China). Obese mice continued on the HFD and were provided *ad libitum* access to water containing an equal-sweetness of MAG (1.1 g/L) or sucrose (266 g/L) for an additional 8 weeks, with a modified protocol from Pino-Seguel et al.^25^ The composition of the experimental diet is listed in Table S1. The dosages of MAG were designed by referencing one previous human randomized controlled trial, in which glycyrrhizin was tested at doses of 1, 2, and 4 mg/kg/day.^20,26^ Moreover, we consider applying the default uncertainty factor of 100 between animal and human dosage; we investigated the effects of MAG on animals at different doses: 100 mg/kg, equivalent to MAG (1.1 g/L) in drinking water. Body weight, food consumption, and water intake were recorded twice a week throughout the experimental period. Oral glucose tolerance tests (OGTTs) were conducted at week 8, before the introduction of MAG, and after 8 weeks of MAG consumption. After the mice were fasted for 12 h, oral gavage was performed using a single dose of MAG (25 mg/kg body weight) followed by oral administration of glucose (2 g/kg body weight) after 30 min of MAG loading. Blood glucose levels were measured at 0, 15, 30, 60, 90, and 120 min after glucose administration by using a Contour Plus glucometer (Bayer, Germany). The area under the curve (AUC) for glucose was calculated using GraphPad Prism 8 software (GraphPad Software, La Jolla, CA, USA). After 8 weeks, the animals were euthanized, and their blood and organs were collected for analysis. Both perirenal and epididymal white adipose tissues were collected as they are representative of visceral fats and are relatively easy to isolate and quantify. Moreover, WAT weights from these sites have been shown to correlate with overall adiposity and metabolic disorders, especially in diet-induced obesity models.^27^

#### Analysis

Blood biochemical analyses were conducted using a Hitachi 7080 Chemistry Analyzer (Hitachi, Tokyo, Japan). The levels of low-density lipoprotein cholesterol (LDL-C) were calculated using the following formula: LDL-C = TC (mg/dL) – HDL-C (mg/dL) – TG (mg/dL)/5, where TC represents total cholesterol, HDL-C represents high-density lipoprotein cholesterol, and TG represents triglyceride. Serum levels of insulin (Mercodia, Uppsala, Sweden), adiponectin, and leptin (BioVendor, Brno, Czechia) were measured using commercial enzyme-linked immunosorbent assay kits following the manufacturer’s instructions.

To explore the potential effects of MAG on adipose tissues and muscles, the mRNA expression levels associated with adipose lipid accumulation and muscle protein turnover were measured using quantitative polymerase chain reaction (qPCR). Briefly, total cellular RNA was isolated from epididymal adipose tissue and gastrocnemius muscle by using an RNeasy Mini Kit (Qiagen, Taipei, Taiwan) and was reverse-transcribed to cDNA by using RevertAid reverse transcriptase (Thermo Fisher Scientific, Cambridge, MA, USA). The resulting cDNA was subsequently used to analyze the target mRNA expression with Maxima SYBR Green/ROX qPCR Master Mix and specific primer sequences (Table S2) in a QuantStudio 1 Real-Time PCR system (Thermo Fisher Scientific). Relative mRNA expression levels were calculated using the comparative C_T_ (2^–ΔΔCt^) method and normalized against internal control GAPDH mRNA levels.

#### Cell Culture Experiments

3T3-L1 preadipocytes (BCRC 60159) were purchased from the Bioresource Collection and Research Center (Hsinchu, Taiwan) and cultured in Dulbecco’s modified Eagle’s medium (DMEM) supplemented with 10% fetal bovine serum at 37 °C in a 5% CO_2_ environment. After incubation in DMEM containing insulin, dexamethasone, and 3-isobutyl-1-methylxanthine (medium A) for 2 days, followed by incubation in an INS-containing medium (medium B) for another 4 days, the preadipocytes differentiated into mature adipocytes.

Commercial glycyrrhizin products available on the food market, including MAG or GL, were used to treat the 3T3-L1 preadipocytes. To determine the effects of MAG or GL on adipogenesis, differentiated mature adipocytes were treated with MAG (20 μM) or GL (20 μM) for 48 h, and the cells were subsequently harvested for analysis. To obtain further insights into the effects of MAG on the differentiation of adipocytes, MAG or GL was added to media A and B, and mature adipocytes were collected on day 6. Oil Red O staining was used to observe the intracellular accumulation of lipids, which is a marker of adipocyte differentiation or adipogenesis (Ramirez-Zacaruas et al.). The intracellular TG content was analyzed using a Randox TRIGS assay kit (Randox Laboratories, Crumlin, UK), and the mRNA expression levels associated with adipocyte differentiation and lipid metabolism were quantified using qPCR as previously described.

### Statistical Analysis

The analysis of variance and chi-square test was employed for continuous and categorical variables, respectively, to compare the demographic data between different Glycyrrhizin consumption groups. Continuous variables were expressed as mean ± standard deviation, and categorical variables were expressed as frequencies and percentages. To determine the longitudinal effect of glycyrrhizin consumption on body composition, linear mixed-effects modeling^28^ was used to analyze repeatedly measured data and determine the correlations between different observations of the same individual at various time points. All analyses were conducted using the lmer test and lme4 packages of R software, version 4.0.3 (R Foundation for Statistical Computing, Vienna, Austria).

Data from the animal and cell experiments are presented as means ± standard deviations. Statistical differences between groups were analyzed using IBM SPSS Statistics, version 19.0 (IBM, Armonk, NY, USA), with one-way analysis of variance (ANOVA) and Tukey’s post hoc test. A *p* value of less than 0.05 was considered significant.

## Results

### Association between Glycyrrhizin Consumption and Body Composition in Children with Obesity and a Normal Weight

In the human study, the TPLS cohort included 1641 children (1111 girls and 530 boys) with data available on both glycyrrhizin consumption and body composition (Table 1; Figure S1). Among these children, 38.67% consumed glycyrrhizin, with 21.51% categorized as high consumers. The average consumption of glycyrrhizin was calculated as 0.12 ± 0.33%ADI. Children in the high-consumption group tended to be younger and less physically active than those in the low-consumption group. The mean BMI *z*-scores and fat mass percentages revealed a significant downward trend across the no-consumption, low-consumption, and high-consumption groups. By contrast, an upward trend was observed in fat-free mass percentage among the three groups.

**Table 1:** Basic Characteristics of the Participants (Taiwan Pubertal Longitudinal Study)^a^

characteristic	no consumption		low consumption		high consumption		total		*p* value
*N*	1007	61.37	281	17.12	353	21.51	1641	100	
age, years	10.24	2.06	10.40	2.21	10.09	2.01	10.23	2.08	<0.01
boys	332	33.00	94	33.69	104	29.55	530	32.38	<0.01
girls	675	38.63	187	66.31	249	70.45	1111	67.62	
sleep quality, PSQI score	4.96	2.45	5.14	2.29	5.00	2.63	5.00	2.46	0.48
total energy intake, kcal	1592.97	452.53	1609.56	475.37	1581.20	445.42	1593.48	454.50	0.59
parental education									0.79
senior high school and lower	69	7.98	13	5.83	22	8.00	104	7.63	
college	496	57.34	133	59.64	157	57.09	786	57.67	
graduate school and higher	300	34.68	77	34.53	96	34.91	473	34.70	
family income, NTD									0.06
<50,000	112	13.02	16	7.17	34	12.45	162	11.95	
50,000–100,000	331	38.49	94	42.15	104	38.10	529	39.01	
>100,000	417	48.49	113	50.67	135	49.45	665	49.04	
physical activity, METD									<0.01
mild (<3)	250	47.71	66	51.16	95	58.28	411	50.37	
moderate (3–6)	125	23.85	30	23.26	31	19.02	186	22.79	
vigorous (>6)	149	28.44	33	25.58	37	22.70	219	26.84	
glycyrrhizin ADI^b^ (%)	0.00	0.00	0.05	0.03	0.50	0.56	0.12	0.33	<0.01
body mass index, kg/m^2^ (*z*-score)	0.32	1.40	0.24	1.41	–0.05	1.34	0.23	1.40	<0.01
obesity^c^	126	12.54	32	11.55	31	8.86	189	11.58	0.06
fat mass (%)	19.90	9.93	19.65	9.60	17.93	9.12	19.47	9.74	<0.01
fat-free mass (%)	80.10	9.93	80.35	9.60	82.07	9.11	80.53	9.74	<0.01
waist-to-height ratio	0.45	0.16	0.44	0.09	0.43	0.08	0.44	0.44	0.39

a The analysis of variance and chi-square test was employed for continuous and categorical variables, respectively, to compare the demographic data between different Glycyrrhizin consumption groups. Data are presented as means ± standard deviations or as numbers (%). PSQI, Pittsburgh Sleep Quality Index; NTD, new Taiwan dollar; METD, Daily Metabolic Equivalent of Task.

b ADI: consumption of glycyrrhizin presented as a percentage of the upper limit of acceptable daily intake. The cutoff between low and high consumption was the median level of glycyrrhizin consumption, which is 0.11%ADI.

c Children with age- and sex-specific body mass index over the 95th percentile were defined as obese in accordance with the standards established by the World Health Organization.

Table 2 presents the differences in body composition (BMI *z*-scores, fat mass percentages, fat-free mass percentages, and waist-to-height ratios) between the glycyrrhizin-consumption groups and the no-consumption group. After adjusting for age, sex, physical activity level, sleep quality, total energy intake, and parental educational level as confounders, we discovered that high consumption of glycyrrhizin was significantly associated with a low BMI *z*-score and fat mass percentage but a high fat-free mass percentage, with significant *p* values. Further categorization of our participants into obese and nonobese groups revealed that the effects of low-fat mass percentage and high-fat-free mass percentage were particularly prominent in the nonobese group.

**Table 2:** Associations between Glycyrrhizin Consumption and Body Composition (Taiwan Pubertal Longitudinal Study)^a^

			BMI *z*-score				fat mass (%)				fat-free mass (%)				waist-to-height ratio			
sweeteners	consumption dose^b^	*N*	beta	95% CI		*p* value^c^	beta	95% CI		*p* value^c^	beta	95% CI		*p* value^c^	beta	95% CI		*p* value^c^
all	no	1007	ref.				ref.				ref.				ref.			
	low	281	–0.05	–0.14	0.04	0.26	–0.51	–1.20	0.19	0.16	0.51	–0.18	1.21	0.15	<0.01	–0.02	0.01	0.57
	high	353	–0.14	–0.23	–0.06	<0.01	–0.80	–1.50	–0.11	0.02	0.81	0.11	1.51	0.02	–0.01	–0.02	0.01	0.39
	*p* trend			<0.01				0.02				0.02				0.35		
nonobese	no	881	ref.				ref.				ref.				ref.			
	low	249	–0.04	–0.13	0.05	0.37	–0.31	–0.95	0.34	0.35	0.31	–0.33	0.96	0.34	–0.01	–0.02	0.01	0.43
	high	322	–0.14	–0.23	–0.06	<0.01	–0.88	–1.51	–0.25	0.01	0.88	0.25	1.52	0.01	<0.01	–0.02	0.01	0.62
	*p* trend			<0.01				0.01				0.01				0.51		
obese	no	126	ref.				ref.				ref.				ref.			
	low	32	–0.07	–0.20	0.06	0.30	–0.39	–2.67	1.89	0.74	0.41	–1.88	2.69	0.73	0.01	–0.02	0.04	0.57
	high	31	–0.09	–0.23	0.05	0.22	–0.28	–2.66	2.09	0.81	0.28	–2.10	2.66	0.82	0.01	–0.02	0.04	0.60
	*p* trend			0.13				0.74				0.74				0.51		

a Exposure dose estimated as proportional to acceptable daily intake and categorized as low or high in the no-consumption group.

b The *p* value presented in the reference row indicates significance for dose responsiveness.

c Models were adjusted for age, sex, physical activity level, sleep quality, total energy intake, and parental educational level.

### Animal Study

After 8 weeks of MAG treatment, the body weight gain and food efficiency ratio of the animals on an AIN-93M basal diet decreased in a dose-dependent manner, although no changes were observed in food or water intake. These effects, particularly at higher concentrations of MAG (3.3 g/L), were accompanied by a decrease in perirenal and epididymal white adipose weight and an increase in relative muscle (gastrocnemius and tibialis anterior) weight (Table 3). In addition, 3.3 g/L MAG lowered the serum levels of leptin and cholesterol, including TC, HDL-C, and LDL-C (Table 4). In animals on an AIN-93M diet, MAG, whether administered in a single dose or over a prolonged period, did not change the levels of blood glucose or the AUC of serum glucose after a 2-h OGTT (Figure S2).

**Table 3:** Effects of MAG on Body and Organ Weight in Animals on an AIN-93M Diet^a^

item/group		control	L-MAG	H-MAG
body weight				
initial (g)		29.3 ± 3.2	30.7 ± 2.0	29.6 ± 2.2
final (g)		35.6 ± 3.9^b^	35.1 ± 3.0^b^	30.9 ± 3.1^a^
change (g)		5.0 ± 3.2^b^	4.4 ± 1.8^b^	1.3 ± 1.2^a^
change (%)		21.5 ± 7.2^c^	14.2 ± 5.5^b^	4.1 ± 3.9^a^
average food intake (g/day)		3.0 ± 0.4	3.1 ± 0.4	3.1 ± 0.5
average water intake (mL/day)		2.4 ± 0.5	2.3 ± 0.7	2.4 ± 1.8
food efficiency ratio (%)^b^		3.5 ± 0.7^c^	2.1 ± 0.9^b^	0.7 ± 0.6^a^
organs or tissues				
heart	(g)	0.14 ± 0.03	0.15 ± 0.03	0.15 ± 0.05
	(%)^c^	0.39 ± 0.08	0.42 ± 0.09	0.49 ± 0.17
liver	(g)	1.19 ± 0.17	1.24 ± 0.15	1.18 ± 0.13
	(%)	3.34 ± 0.24^a^	3.53 ± 0.27^ab^	3.84 ± 0.44^b^
spleen	(g)	0.08 ± 0.04	0.09 ± 0.03	0.1 ± 0.03
	(%)	0.24 ± 0.12	0.25 ± 0.08	0.32 ± 0.1
kidneys	(g)	0.38 ± 0.04	0.38 ± 0.04	0.36 ± 0.06
	(%)	1.09 ± 0.1	1.07 ± 0.1	1.15 ± 0.15
white adipose tissue				
perirenal (P)	(g)	0.69 ± 0.25^b^	0.62 ± 0.18^ab^	0.38 ± 0.12^a^
	(%)	1.92 ± 0.59^b^	1.76 ± 0.49^ab^	1.24 ± 0.37^a^
epididymal (E)	(g)	1.37 ± 0.54^b^	1.26 ± 0.35^ab^	0.85 ± 0.3^a^
	(%)	3.84 ± 1.34^b^	3.57 ± 0.85^ab^	2.74 ± 0.88^a^
P + E	(g)	2.06 ± 0.73^b^	1.87 ± 0.51^b^	1.23 ± 0.41^a^
	(%)	5.76 ± 1.75 ^b^	5.33 ± 1.28 ^ab^	3.98 ± 1.2 ^a^
muscle				
gastrocnemius (GA)	(g)	0.34 ± 0.03	0.33 ± 0.05	0.34 ± 0.05
	(%)	0.97 ± 0.1^a^	0.95 ± 0.13^a^	1.11 ± 0.15^b^
tibialis anterior (TA)	(g)	0.13 ± 0.02	0.12 ± 0.01	0.13 ± 0.03
	(%)	0.35 ± 0.04^ab^	0.33 ± 0.04^a^	0.42 ± 0.09^b^
GA + TA	(g)	0.47 ± 0.04	0.45 ± 0.06	0.47 ± 0.05
	(%)	1.32 ± 0.12^a^	1.28 ± 0.14^a^	1.53 ± 0.16^b^

a L-MAG (1.1 g/L) or H-MAG (3.3 g/L) was administered to the animals in their drinking water for 8 weeks, followed by euthanasia and organ collection. Values are presented as means ± standard deviations (*n* = 8–10). Differences between groups were determined using one-way ANOVA followed by Tukey’s post hoc test. ^a–c^Values with different superscripts are significantly different (*p* < 0.05). MAG, monoammonium glycyrrhizinate.

b Food efficiency ratio = (body weight gain (g)/food intake (g)) × 100%.

c Relative organ weight = (organ weight (g)/body weight (g)) × 100%

**Table 4:** Effects of MAG on Blood Biochemical Measurements in Animals on an AIN 93M Diet^a^

item/group	control	L-MAG	H-MAG
AST (U/L)	99.0 ± 21.8	98.5 ± 31.2	118.7 ± 28.1
ALT (U/L)	37.7 ± 20.3	22.0 ± 7.3	24.3 ± 13.0
glucose (mg/dL)	175.3 ± 67.7	150.2 ± 61.4	165.8 ± 42.9
BUN (mg/dL)	15.2 ± 3.1	16.6 ± 3.2	19.0 ± 2.7
creatinine (mg/dL)	0.1 ± 0.02	0.10 ± 0.04	0.1 ± 0.01
TC (mg/dL)	160.1 ± 17.0^b^	140.6 ± 24.9^ab^	120.0 ± 23.0^a^
TG (mg/dL)	36.1 ± 12.9	49.8 ± 39.7	47.5 ± 30.9
HDL-C (mg/dL)	126.3 ± 11.4^b^	109.3 ± 18.3^ab^	95.8 ± 18.8^a^
LDL-C (mg/dL)	22.0 ± 3.6^b^	20.4 ± 5.8^ab^	14.6 ± 4.3^a^
insulin (μg/L)	1.7 ± 2.2	0.9 ± 0.6	1.0 ± 0.6
adiponectin (ng/mL)	28.4 ± 14.8	31.5 ± 20.2	32.4 ± 13.5
leptin (μg/mL)	8.7 ± 4.0^b^	5.2 ± 2.4^ab^	2.7 ± 1.8^a^

a L-MAG (1.1 g/L) or H-MAG (3.3 g/L) was administered to the animals in their drinking water for 8 weeks, and blood samples were collected for analysis. Values are presented as means ± standard deviations (*n* = 8–10). Differences between groups were determined using one-way ANOVA followed by Tukey’s post hoc test. ^ab^Values with different superscripts are significantly different (*p* < 0.05). MAG, monoammonium glycyrrhizinate; AST, aspartate transaminase; ALT, alanine aminotransferase; TC, total cholesterol; TG, triglyceride; HDL-C, high-density lipoprotein cholesterol; LDL-C, low-density lipoprotein cholesterol.

**Figure 1:**
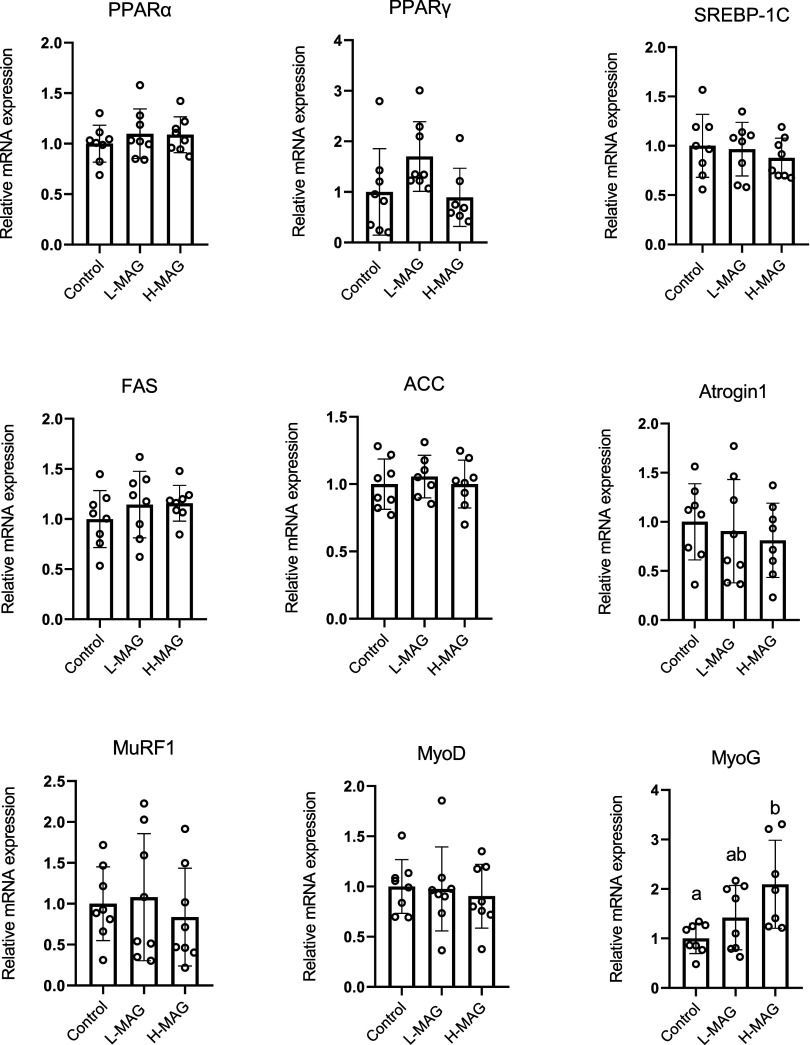
**Effects of MAG on the mRNA expression of adipogenesis-associated factors of white adipose tissue and muscle protein-turnover factors in animals fed with an AIN-93M diet.** The animals were administered with L-MAG (1.1 g/L) or H-MAG (3.3 g/L) in drinking water for 8 weeks. After sacrifice, the epididymal adipose tissue and gastrocnemius muscle were collected and subjected to analysis of mRNA expression by qPCR. Values are expressed as mean ± SD (*n* = 8). Differences between groups were determined by one-way ANOVA followed by the post hoc Tukey test. ab, Data do not share the same letter significantly differ (*p* < 0.05). qPCR, quantitative polymerase chain reaction. PPAR, peroxisome proliferator-activated receptor; FAS, fatty acid synthase; ACC, Acyl-CoA synthase; MyoG, myogenin; MyoD, myogenic differentiation

Unlike in the healthy animals, in the HFD-induced obese mice, administering MAG for 8 weeks did not affect body weight gain, although the sucrose group exhibited a higher body weight gain than that of the HFD group, presumably because of their higher sucrose consumption and food efficiency ratio. As indicated in Tables 5 and 6, neither MAG nor sucrose affected the organ weights or blood biochemical measurements of the obese mice. Similar to exposure to sucrose, prolonged exposure to MAG (8 weeks) significantly increased the AUC of blood glucose, although single exposure to MAG had no effect (Figure S3). These findings indicate that MAG may have an effect on the body composition and lipid metabolism of healthy animals. Nevertheless, prolonged exposure to MAG does not affect adiposity and may deteriorate glucose intolerance in obese animals.

**Table 5:** Effects of MAG on Body and Organ Weight in HFD-Fed Obese Mice^a^

item/group		HFD	sucrose	MAG
body weight				
initial (g)		36.6 ± 3.4	35.8 ± 4.5	36.9 ± 3.5
final (g)		42.3 ± 2.2	45.9 ± 4.3	44.8 ± 2.2
change (g)		5.7 ± 2.6^a^	10.1 ± 3.6^b^	8.0 ± 1.7^ab^
change (%)		16.1 ± 8.3^a^	29.1 ± 12.2^b^	22.2 ± 6.5^ab^
average food intake (g/day)		2.4 ± 0.2	2.6 ± 1.5	2.5 ± 0.2
average water intake (mL/day)		2.2 ± 0.7^a^	6.8 ± 3.7^b^	1.8 ± 0.4^a^
food efficiency ratio (%)^b^		3.5 ± 1.5^a^	7.6 ± 2.6^b^	4.4 ± 1.0^a^
organs or tissues				
heart	(g)	0.17 ± 0.06	0.16 ± 0.03	0.16 ± 0.02
	(%)^c^	0.42 ± 0.13	0.36 ± 0.08	0.35 ± 0.04
liver	(g)	1.17 ± 0.17	1.43 ± 0.27	1.34 ± 0.04
	(%)	2.90 ± 0.36	3.14 ± 0.60	2.99 ± 0.56
spleen	(g)	0.10 ± 0.05	0.09 ± 0.02	0.09 ± 0.02
	(%)	0.25 ± 0.12	0.20 ± 0.06	0.19 ± 0.05
kidneys	(g)	0.39 ± 0.08	0.39 ± 0.04	0.40 ± 0.03
	(%)	0.98 ± 0.21	0.87 ± 0.17	0.88 ± 0.07
white adipose tissue				
perirenal (P)	(g)	0.89 ± 0.26	1.17 ± 0.31	1.21 ± 0.19
	(%)	2.30 ± 0.64	2.53 ± 0.54	2.71 ± 0.43
epididymal (E)	(g)	1.88 ± 0.26	2.09 ± 0.39	2.11 ± 0.40
	(%)	4.86 ± 0.84	4.55 ± 0.71	4.72 ± 0.97
P + E	(g)	2.77 ± 0.43	3.26 ± 0.62	3.32 ± 0.43
	(%)	7.17 ± 1.23	7.07 ± 0.99	7.43 ± 1.08
muscle				
gastrocnemius (GA)	(g)	0.32 ± 0.05	0.34 ± 0.04	0.36 ± 0.06
	(%)	0.88 ± 0.12	0.75 ± 0.11	0.81 ± 0.15
tibialis anterior (TA)	(g)	0.13 ± 0.02	0.12 ± 0.02	0.13 ± 0.01
	(%)	0.35 ± 0.06	0.27 ± 0.05	0.29 ± 0.04
GA + TA	(g)	0.45 ± 0.05	0.47 ± 0.04	0.49 ± 0.06
	(%)	1.11 ± 0.09	1.02 ± 0.14	1.10 ± 0.14

a After the animals received an HFD for 8 weeks, MAG (1.1 g/L) or equivalent-sweetness sucrose (266 g/L) was added to their drinking water for another 8 weeks. Subsequently, the animals were euthanized, and their organs were collected. Values are presented as means ± standard deviations (*n* = 8–10). Differences between groups were determined using one-way ANOVA followed by Tukey’s post hoc test. ^ab^Values with different superscripts are significantly different (*p* < 0.05). MAG, monoammonium glycyrrhizinate; HFD, high-fat diet.

b Food efficiency ratio = (body weight gain (g)/food intake (g)) × 100%.

c Relative organ weight = (organ weight (g)/body weight (g)) × 100%.

**Table 6:** Effects of MAG on Blood Biochemical Measurements in HFD-Fed Obese Mice[Table-fn t6fn1]

item/group	control	sucrose	MAG
AST (U/L)	131.0 ± 17.7	139.9 ± 40.5	146.2 ± 47.1
ALT (U/L)	89.5 ± 20.3	115.4 ± 70.6	140.9 ± 90.6
glucose (mg/dL)	198.6 ± 9.4	154.3 ± 48.1	160.3 ± 29.2
BUN (mg/dL)	15.2 ± 3.1	16.6 ± 3.2	19.0 ± 2.7
creatinine (mg/dL)	0.1 ± 0.02	0.1 ± 0.04	0.1 ± 0.01
TC (mg/dL)	143.2 ± 45.8	165.6 ± 40.3	152.5 ± 53.5
TG (mg/dL)	32.2 ± 3.0	31.6 ± 13.9	26.4 ± 12.9
HDL-C (mg/dL)	110.7 ± 11.4	121.6 ± 29.2	113.3 ± 34.9
LDL-C (mg/dL)	26.1 ± 14.8	37.7 ± 15.3	33.9 ± 17.3
insulin (μg/L)	1.7 ± 1.1	1.3 ± 0.9	1.5 ± 1.1
adiponectin (ng/mL)	43.5 ± 15.3	23.4 ± 11.4	38.3 ± 21.5
leptin (μg/mL)	17.1 ± 13.2	20.6 ± 11.3	21.6 ± 11.8

1 After the animals received an HFD for 8 weeks, MAG (1.1 g/L) or equivalent-sweetness sucrose (266 g/L) was added to their drinking water for another 8 weeks. Subsequently, blood samples were collected for analysis. Values are presented as means ± standard deviations (*n* = 8–10). Differences between groups were determined using one-way ANOVA followed by Tukey’s post hoc test. ^ab^Values with different superscripts are significantly different (*p* < 0.05). MAG, monoammonium glycyrrhizinate; AST, aspartate transaminase; ALT, alanine aminotransferase; TC, total cholesterol; TG, triglyceride; HDL-C, high-density lipoprotein cholesterol; LDL-C, low-density lipoprotein cholesterol; HFD, high-fat diet.

To identify the potential mechanism underlying the effect of MAG on healthy animals, we analyzed the mRNA expression of adipogenesis- or muscle-protein-related factors in epididymal adipose tissue and gastrocnemius muscle, respectively. As shown in Figure 1, we observed no changes in the mRNA expression of adipogenesis-related factors, including peroxisome proliferator-activated receptor-α (PPAR-α), PPAR-γ, SREBP-1c, fatty acid synthase (FAS), and acyl-CoA carboxylase. We also observed no changes in the muscle protein degradation factors Atrogin-1 and MuRF-1 or the muscle protein synthesis factor MyoD. At a higher concentration, MAG upregulated the mRNA expression of MyoG, which is a muscle protein synthesis factor (Figure 1).

### Cultured Cells

After mature adipocytes were treated with MAG or GL for 2 days, no change was observed in their lipid accumulation (Figures 2A and 2B). However, when media A and B were added during the preadipocyte differentiation stage, a significant reduction was observed in their intracellular lipid and TG accumulation (Figures 2C and 2D), with no effect on cell viability (data not shown). These findings are consistent with those of the human and animal studies, in which MAG had no effect on obese animals but reduced the fat mass percentage of nonobese animals.

**Figure 2:**
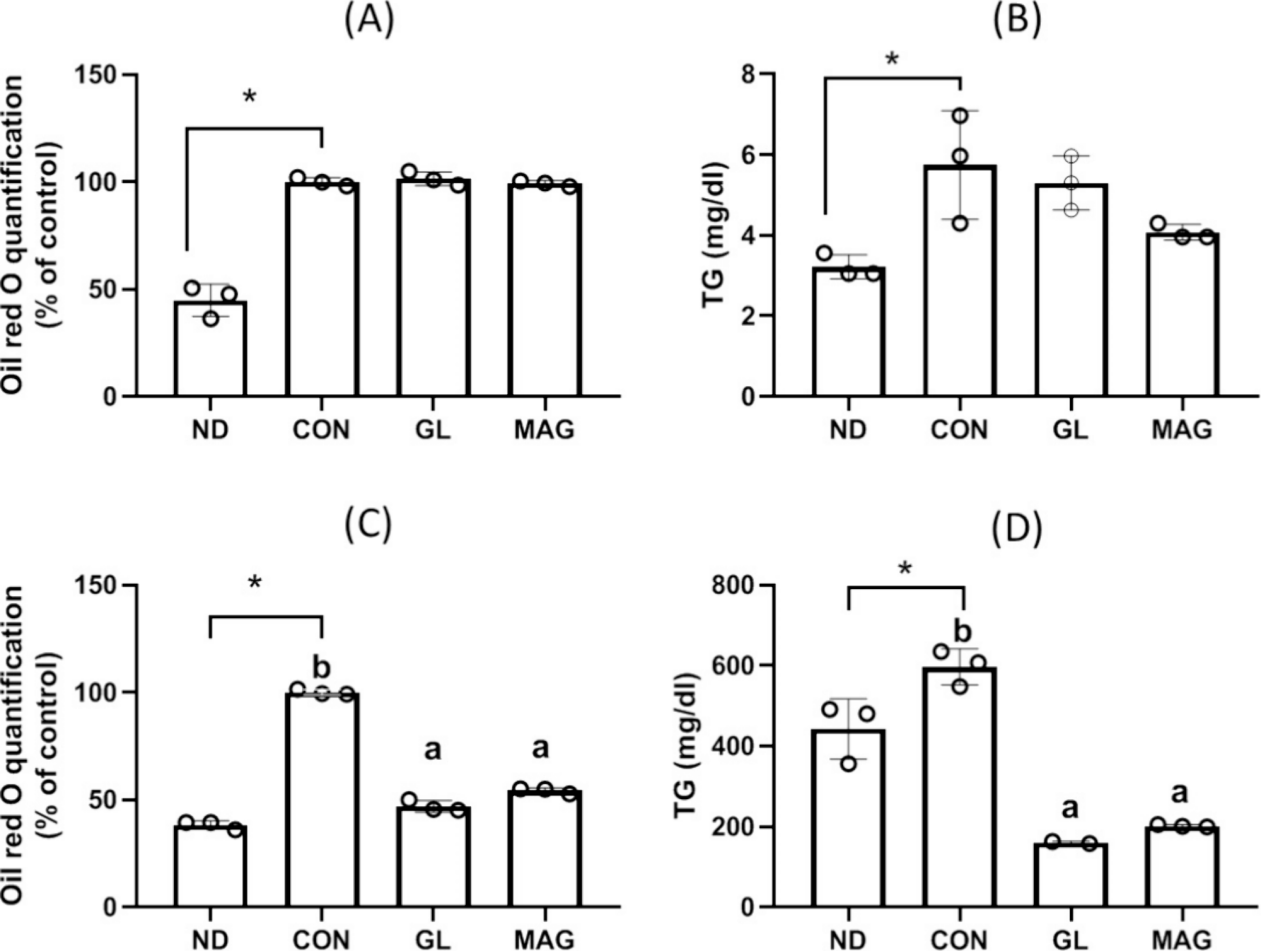
**Effects of MAG on the oil red quantification (A, C) and triglyceride accumulation (B, D) in mature adipocytes.** MAG (20 μM) or GL (20 μM) was added to the 3T3-L1 mature adipocytes for 48 h (A, B), or added in the 3T3-L1 preadipocyte differentiating medium for 6 ds (C, D), the lipid accumulation in adipocytes were determined by oil red O staining and quantification or triglyceride contents. Values are expressed as mean ± SD (*n* = 8). Differences between groups were determined by one-way ANOVA followed by the post hoc Tukey test. *, significantly different from the ND group. ab, Data do not share the same letter significantly differ among differentiated adipocytes (*p* < 0.05). qPCR, quantitative polymerase chain reaction; ND, nondifferentiated preadipocyte; CON, adipocyte control; GL glycyrrhizin, MAG, monoammonium glycyrrhizinate.

To gain further insights into the inhibitory effects of MAG and GL on adipocyte differentiation, we analyzed the mRNA expression of various differentiation-associated markers. Similar to lipid accumulation, MAG and GL downregulated the mRNA expression of mature adipocyte markers such as GLUT4, leptin, and adiponectin. GL also inhibited the expression of C/EBPα, a transcription factor involved in the differentiation of adipocytes, indicating a potential inhibitory role of GL, and presumably MAG, in preadipocyte differentiation. In addition, MAG and GL inhibited the expression of the adipocyte browning markers UCP1 and PRDM16 but did not affect the expression levels of the sweet taste receptors T1R2 and T1R3 (Figure 3). These findings suggest that MAG not only reduces the accumulation of fat in healthy individuals but also inhibits differentiation of adipocytes. In individuals with obesity, MAG does not exert an inhibitory effect because these individuals have a large number of differentiated mature adipocytes.

**Figure 3:**
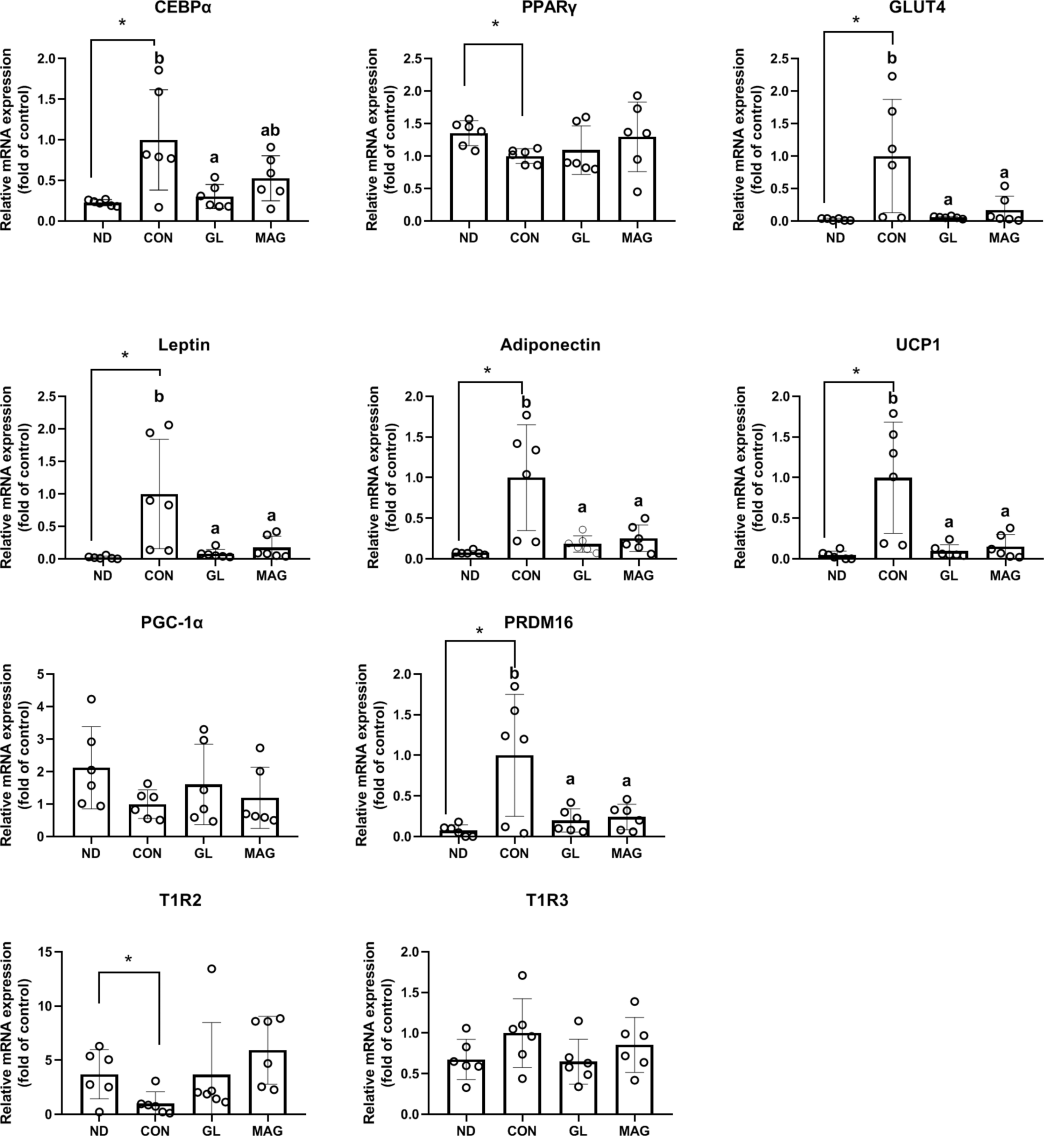
**Effects of MAG and GL on the mRNA expression of adipocyte differentiation-associated factors as indicated.** 3T3-L1 preadipocytes were treated with MAG (20 μM) or GL (20 μM) throughout the differentiation period for 6 days. The mRNA expression in differentiated adipocytes was analyzed by qPCR. Values are expressed as mean ± SD (*n* = 6). Differences between ND and CON were analyzed by Student’s *t* test, and differences between the CON and the MAG or the GL group were analyzed by one-way ANOVA followed by Tukey post hoc test. *, significantly different from the ND group. ab, Data do not share the same letter significantly differ among differentiated adipocytes (*p* < 0.05). qPCR, quantitative polymerase chain reaction; ND, nondifferentiated preadipocyte; CON, adipocyte control; GL glycyrrhizin, MAG, monoammonium glycyrrhizinate C/EBPα, CCAAT-enhancer-binding protein α; PPAR-γ, peroxisome proliferator-activated receptor-γ; GLUT4, glucose transporter 4; UCP1, uncoupling protein 1; PGC-1α, peroxisome proliferator-activated receptor gamma coactivator −1α; PRDM16, PR domain containing 16; SREBP-1c, sterol regulatory element-binding protein-1c; ACC, acetyl-CoA carboxylase; FAS, fatty acid synthase; T1R2, taste 1 receptor member 2; T1R3, taste 1 receptor member 3.

## Discussion

In our *in vivo* and *in vitro* experiments, we discovered that glycyrrhizin not only reduces fat accumulation but also increases muscle mass. Glycyrrhizin exerts an adiposity-reducing effect in nonobese but not in obese individuals, as indicated by our human and animal studies. During the preadipocyte differentiation stage, glycyrrhizin reduces the accumulation of intracellular lipids and TGs, but this effect is not observed in mature adipocytes. Overall, we discovered that glycyrrhizin lowers the degree of adiposity by inhibiting differentiation of adipocytes, as indicated by the significant reduction observed in the mRNA expression levels of C/EBPα, GLUT4, leptin, and adiponectin. In the current NNS-focused era, glycyrrhizin can be used as a commercial substitute for sugar. Further research is required to determine whether the adiposity-reducing and muscle-restoring effects of glycyrrhizin can be useful in treatment for children under rapid pubertal growth.

According to our human and animal studies, glycyrrhizin can be used as an adiposity-reducing agent. To the best of our knowledge, this is the first human study to elucidate the adiposity-reducing effect of glycyrrhizin on nonobese adolescents. We used various animal models to validate the findings from our human cohort. This adiposity-reducing effect (perirenal, epididymal) was observed only in mice on a normal diet and not in HFD-fed mice. An improvement in metabolic profiles, including TC, TG, and LDL, was also observed only in mice on a normal diet. Previous animal studies have indicated that glycyrrhizin reduces fat accumulation. Abo El-Magd et al.^12^ orally gavaged 50 mg/kg/day glycyrrhizin to obese Wistar rats for 4 weeks and reported a significant reduction in abnormal weight gain, insulin resistance, adipose tissue cell size, and liver fat accumulation. Sil et al.^29^ gave a single intraperitoneal injection of glycyrrhizin (50 mg/kg) to Wistar rats on a high-fructose diet for 2 weeks and reported a significant reduction in abnormal weight, blood glucose levels, blood TG levels, and liver oxidative stress associated with metabolic syndrome. In the present study, MAG, rather than glycyrrhizin was applied. Both glycyrrhizin and MAG are approved sweetener additives in Taiwan. Because of the higher sweetness and water-soluble characteristics, MAG is more prevalently used in the industry. In addition, the discrepancy between our animal experiments and those conducted in previous studies may be due to the different time durations and administration routes of glycyrrhizin and the different species of animals used. Nonetheless, Lee et al.^30^ conducted cell experiments involving a glycyrrhizin-containing licorice root extract (≤0.5% w/w) to explore the differentiation process of 3T3-L1 preadipocytes. Consistent with our findings, they discovered that this extract regulated the third stage of cell differentiation (i.e., clonal expansion) and controlled the levels of adenosine monophosphate-activated protein kinase (AMPK), thereby inhibiting lipogenesis.

In the present study, we discovered that MAG and GL significantly reduced the intracellular accumulation of lipids and TGs during the preadipocyte differentiation process. According to Darlington et al.,^8^ C/EBPα plays a key role in the early differentiation of adipocytes by not only activating adipocyte-specific genes such as GLUT4, thus sustaining differentiation, but also serving as a primary regulatory factor in intracellular TG accumulation. In a cell biology study, Wu et al.^31^ discovered significantly reduced lipid accumulation in the preadipocytes of C/EBPα^–/–^ mice after differentiation, along with reduced expression of another key transcription factor, PPAR-γ, and certain adipocyte-specific genes such as adipsin, FAS, and lipoprotein lipase. However, upon the transfection and expression of the C/EBPα gene in the cells, the accumulation of lipids increased, and the expression of the differentiation marker PPAR-γ and certain adipocyte-specific genes increased. These findings indicate the essential role of C/EBPα in early differentiation of adipocytes. In this study, we discovered that, during the 6-day differentiation process, downregulating the gene expression of C/EBPα presumably inhibited the differentiation of adipocytes and the intracellular accumulation of lipids. In a previous study, Yamamoto et al.^32^ discovered that glycyrrhetinic acid (GA) inhibited differentiation of 3T3-L1 adipocytes during the 6-day differentiation process, reducing intracellular accumulation of lipids and the levels of the early differentiation markers C/EBPβ and C/EBPδ. Nevertheless, simultaneous administration of GA and MEK inhibitors significantly downregulated the gene and protein expression of C/EBPβ and C/EBPδ, thereby inhibiting early differentiation. In another study, Lee et al.^30^ discovered that a licorice root extract containing GL and other active components, including MAG, inhibited differentiation of 3T3-L1 adipocytes, with a significant increase noted in the expression of AMPK, suggesting that its inhibitory effect on cell differentiation is associated with AMPK activation. These findings suggest that GL and MAG inhibit the differentiation of adipocytes by activating AMPK and the MEK/ERK pathway.

In this study, we conducted a 3T3-L1 cell differentiation process to determine the physiological mechanisms underlying the stronger adiposity-reducing effects of glycyrrhizin in nonobese individuals than in obese ones. Our findings indicate that GL and MAG, when treating the mature adipocytes that have undergone completion of differentiation, which mimics the adipocytes in the obese individuals, showed no significant impact on lipid accumulation after two days. However, when added in the medium throughout the differentiation period, GL and MAG decreased the lipid accumulation in the mature adipocytes, which resembles the preadipocyte differentiation in nonobese subjects. Consistent with the results of previous study, licorice acetone extract (LE) and GL have been shown to suppress adipogenesis by interfering the differentiation process, because when GA was treated in the mid (second to fourth day) or late (fourth to sixth day) stages of 3T3-L1 adipocyte differentiation, no effect on lipid accumulation in the cells was observed,^30,32^ consistent with our findings. This phenomenon occurred presumably because the key transcription factors C/EBPα and PPAR-γ, which are involved in adipocyte differentiation, were activated early during the differentiation process, thereby inducing expression of certain adipocyte-specific genes and lipid-metabolism-related genes and triggering accumulation of TGs in the cells. Because the effects of GL and MAG on adipocytes are often observed early during the differentiation process, interventions during the intermediate or late stage may not be associated with significant inhibitory effects.

Contrary to our expectations, we discovered that MAG had both an adiposity-reducing effect and a muscle-restoring effect. In nonobese children and mice fed with the AIN-93M diet, the fat-free mass percentage of the children (Table 2) and the muscle organ weight of the mice significantly increased with the MAG dosage (Table 3). The mRNA expression level of MyoG (myogenic gene) also significantly increased with the MAG dosage (Figure 1). Several studies have reported similar findings. In a Korean study, Lee et al.^33^ reported that pretreatment of muscle injury mice with glycyrrhizin enhanced their muscle regeneration by inducing expression of MyoGs. In a mouse model of amyotrophic lateral sclerosis, Cai et al.^34^ discovered that an herbal mixture containing glycyrrhizin influenced motor function and had a protective effect against muscle denervation. In a Chinese study, Li et al.^35^ reported that glycyrrhizin polysaccharides significantly upregulated the expression of MyoG and MyoD mRNA in the leg muscles of animal models.

This study has several strengths. For example, we obtained consistent results from our *in vivo* and *in vitro* experiments, indicating the translational value. Our findings also confirmed that glycyrrhizin has not only an adiposity-reducing effect but also a muscle-restoring effect on both humans and mice. In addition, we discovered that the effect of glycyrrhizin on 3T3-L1 cells is specifically observed during the preadipocyte differentiation stage; it is not observed in mature adipocytes. Despite these strengths, this study has several limitations. First, we conducted an observational cohort study instead of a human interventional trial, and this might preclude causal inference. Second, the limited sample size of the obese population within the TPLS cohort and unmeasured confounders may contribute to inconclusive results. Third, although we used a validated food frequency questionnaire, the dietary intake of glycyrrhizin was self-reported and may be subject to recall bias. Fourth, the glycyrrhizin dosages used in the human, animal, and cellular experiments were not uniform and may differ in bioavailability across species.

In summary, glycyrrhizin has adiposity-reducing and muscle-restoring effects on nonobese children and mice. These effects are induced by inhibition of adipocyte differentiation during the preadipocyte differentiation stage but not during the adipocyte maturation stage. These findings indicate that glycyrrhizin may favorably modulate body composition in nonobese individuals by attenuating adipocyte differentiation and supporting muscle growth. However, its preventive or therapeutic role in obesity remains to be clarified through clinical trials. Further clinical intervention studies are required to determine the effects of glycyrrhizin on children among pubertal growing children and older patients with sarcopenia.

## Supplementary Material



## Data Availability

Data described in this manuscript, code book, and analytical code will be shared with other researchers for further analysis upon contacting the authors.

## Notes

Table S1. Composition of the experimental diet. Table S2. Primer sequences used in RT-PCR. Figure S1. Study Flow of Taiwan Pubertal Longitudinal Study. Figure S2. Effects of MAG on blood glucose levels and area under the curve (AUC) of serum glucose from OGTT after a single dose (A) or after 8-week exposure (B) in mice fed with an AIN-93M diet. Figure S3. Effects of MAG and sucrose on blood glucose levels and area under the curve (AUC) of serum glucose from OGTT after a single dose (A) or after 8-week exposure (B) in HFD-induced obese mice. (PDF)

## Abbreviations

ACC: Acyl-CoA carboxylase
ADI: acceptable daily intake
AUC: area under the curve
ANOVA: one-way analysis of variance
BMI: body mass index
C/EBPα: CCAAT/enhancer-binding protein alpha
GL: glycyrrhizic acid
GLUT4: glucose transporter type 4
HFDs: high-fat diets
HOMA-IR: homeostatic model assessment for insulin resistance
LDL-C: low-density lipoprotein cholesterol
MAG: monoammonium glycyrrhizinate
METD: Daily Metabolic Equivalent of Task
MyoG: myogenin
MyoD: myogenic differentiation factor
FAS: fatty acid synthase
FFQ: Food Frequency Questionnaire
NNS: non-nutritive sweeteners
OGTTs: oral glucose tolerance tests
PGC-1α: peroxisome proliferator-activated receptor gamma coactivator-1 alpha
PPAR: peroxisome proliferator-activated receptor
PRDM16: PR domain containing 16
SREBP-1c: sterol regulatory element-binding protein-1c
TPLS: Taiwan Puberty Longitudinal Study
qPCR: quantitative polymerase chain reaction
UCP1: uncoupling protein 1
T1R2/T1R3: taste receptor type 1 members 2 and 3
