# Supplementary material for: Body Recomposition Effects of Long-Term Glycyrrhizin Consumption in Nonobese Individuals: From the Clinic to the Bench
Source: ACS Pharmacol Transl Sci. 2025 Jul 15;8(8):2536–47. doi: 10.1021/acsptsci.5c00120 (PMC12340619; doi:10.1021/acsptsci.5c00120)
Supplement: Supplementary file 1 [file pt5c00120_si_001.pdf]

Supporting Information

Body Recomposition Effects of Long-Term Glycyrrhizin Consumption in Nonobese Individuals: From the Clinic to the Bench

Yang-Ching Chen[a,b,c,d,e], Yu-Cih Huang[c#], Yu-Jie Cheng[c#], Jessika Woo Kar Man[c], Rong-Hong Hsieh[c], Shih-Yuan Hsu[b], Yue-Hwa Chen[c,f,g*]

[a]Department of Family Medicine, Wan Fang Hospital, Taipei Medical University, Taipei, 116, Taiwan

[b]Department of Family Medicine, School of medicine, College of medicine, Taipei Medical University, Taipei, 110, Taiwan

[c]School of Nutrition and Health Sciences, College of Nutrition, Taipei Medical University, Taipei, 110, Taiwan

[d]Graduate Institute of Metabolism and Obesity Sciences, Taipei Medical University, Taipei, 110, Taiwan

[e]Nutrition Research Center, Taipei Medical University Hospital, Taipei, 110, Taiwan

[f]School of Food Safety, College of Nutrition, Taipei Medical University, Taipei, 110, Taiwan

[g]Research Center of Food Safety Inspection and Function Development, College of Nutrition, Taipei Medical University, Taipei, 110, Taiwan

#equally contributed

*Yue-Hwa Chen, Ph.D. Email: yuehwa@tmu.edu.tw

TABLE OF CONTENTS

Table S1. Composition of the experimental diet (kg) **3**

Table S2. Primer sequences used in RT-PCR. **4**

Figure S1. Study Flow of Taiwan Pubertal Longitudinal Study **5**

Figure S2. Effects of MAG on blood glucose levels and area under the curve (AUC) of serum glucose from OGTT after a single dose (A) or after 8-week exposure (B) in mice fed with an AIN-93M diet. The OGTT was performed before or after 8-week administration of L-MAG (1.1 g/L) or H-MAG (3.3 g/L) in drinking water, respectively. **6**

Figure S3. Effects of MAG and sucrose on blood glucose levels and area under the curve (AUC) of serum glucose from OGTT after a single dose (A) or after 8-week exposure (B) in HFD-induced obese mice. **7**

Table S1. Composition of the experimental diet (kg)

	AIN-93M	HFD
**Ingredient**	**g**	**g**
**Casein**	140	130
**Soybean oil**	40	24
**Lard**	-	338
**AIN-93 mineral**	35	35
**AIN-93 vitamin**	10	10
**Corn starch**	621	279
**Cellulose**	50	50
**Sucrose**	100	130
**L-Cystine**	1.8	1.8
**Choline bitartrate**	2.5	2.5
**t-Butyl hydroquinone**	0.008	0.008
**Total**	1000	1000
**Protein**	15%	10%
**Fat**	10%	60%
**Carbohydrate**	75%	30%

Casein, Soybean oil, Lard, AIN-93 mineral, AIN-93 vitamin, Corn starch, Cellulose, Sucrose, L-Cystine, Choline bitartrate, and t-Butyl hydroquinone were purchased from MP Biomedicals, Irvine, CA, USA.

Table S2. Primer sequences used in RT-PCR.

Gene	Forward (5'→3')	Reverse (5'→3')	Accession No.[a]
β-actin	TGTCCACCTTCCAGCAGATGT	AGCTCAGTAACAGTCCGCCTA GA	X03672
C/EBPα	CGCAAGAGCCGAGATAAAGC	CACGGCTCAGCTGTTCCA	NM_001287514.1
PPAR-γ	CAGCAGGTTGTCTTGGATGTC	AGCCCTTTGGTGACTTTATGG	XM_029535224.1
GLUT4	GACGGACACTCCATCTGTTG	GCCACGATGGAGACATAGC	NM_009204
Leptin	TCTGAAAGATCCCACGTGCC	AAGGCTCAGGACATTCCAGC	NM_008493.3
Adiponectin	ATCTGGAGGTGGGAGACCAA	GGGCTATGGGTAGTTGCAGT	NM_009605.5
IL-6	TCCTACCCCAACTTCCAATGCTC	TTGGATGGTCTTGGTCCTTAG CC	XM_032905335.1
UCP1	CCTGCCTCTCTCGGAAACAA	GTAGCGGGGTTTGATCCCAT	NM_009463.3
PGC-1α	TATGGAGTGACATAGAGTGTGCT	CCACTTCAATCCACCCAGAAA G	NM_008904.3
PRDM16	CCACCAGCGAGGACTTCAC	GGAGGACTCTCGTAGCTCGAA	XM_045151135.1
SREBP-1c	CACTTCTGGAGACATCGCAAAC	ATGGTAGACAACAGCCGCATC	NM_001358315.1
ACC	ATGGGCGGAATGGTCTCTTTC	TGGGGACCTTGTCTTCATCAT	NM_133360.3
FAS	GGAGGTGGTGATAGCCGGTAT	TGGGTAATCCATAGAGCCCAG	XM_030245556.1
ATGL	GGATGAAAGAGCAGACGGGTAG	CGCAAGACAGTGGCACAGAG	NM_025802.3
HSL	ACTGAGATTGAGGTGCTGTC	AGGTGAGATGGTAACTGTGAG	NM_001039507.2
MGL	TCGGAACAAGTCGGAGGT	TCAGCAGCTGTATGCCAAAG	XM_031382510.1

[a] NCBI GenBank database.

C/EBPα, CCAAT-enhancer-binding protein α; PPAR-γ, peroxisome proliferator-activated receptor-γ; GLUT4, glucose transporter 4; UCP1, uncoupling protein 1; PGC-1α, peroxisome proliferator-activated receptor gamma coactivator -1α; PRDM16, PR domain containing 16; SREBP-1c, sterol regulatory element-binding protein-1c; ACC, acetyl-CoA carboxylase; FAS, fatty acid synthase; ATGL, adipose triglyceride lipase; HSL, hormone-sensitive lipase; MGL, monoacylglycerol lipase; IL-6, interleukin 6

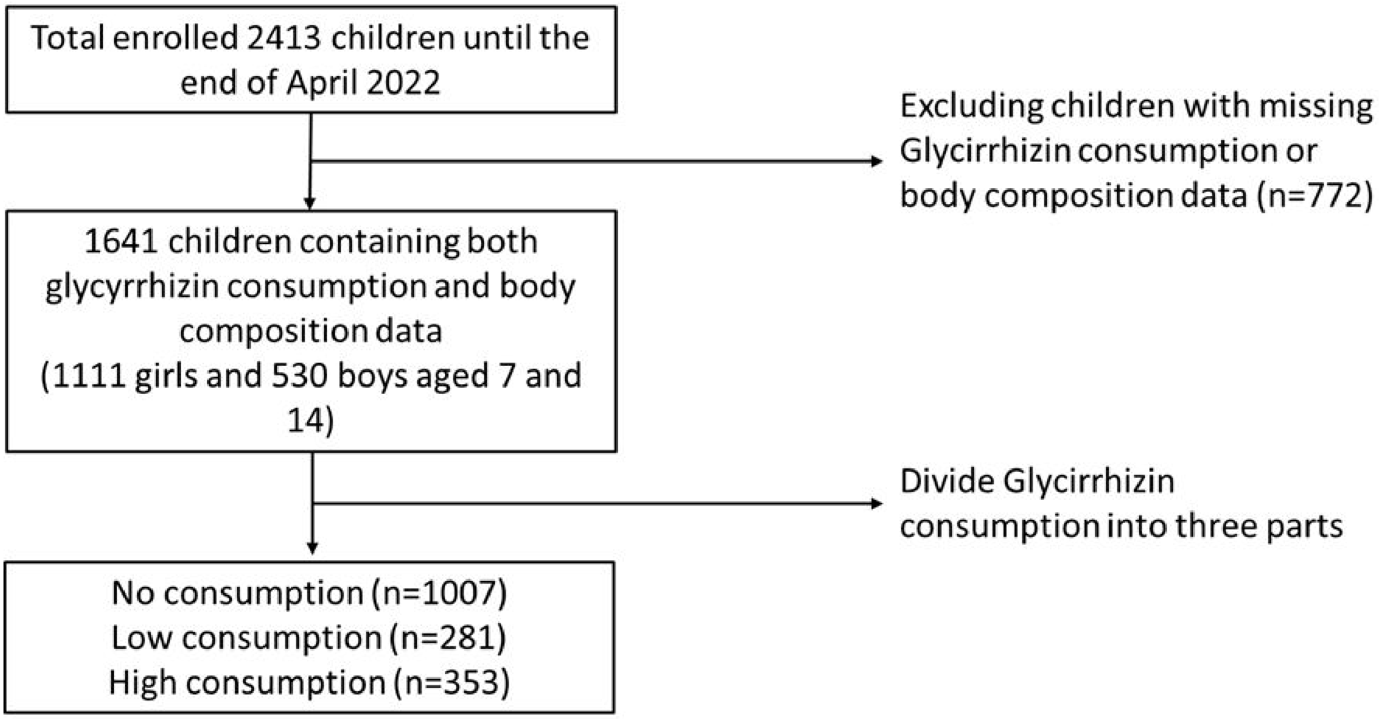

Figure S1. **Study Flow of Taiwan Pubertal Longitudinal Study**

(A)

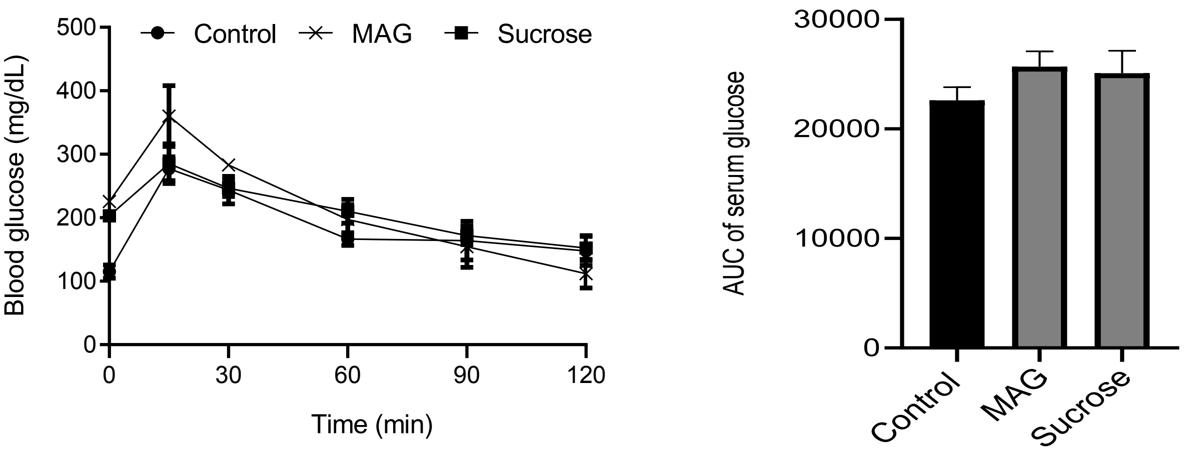

(B)

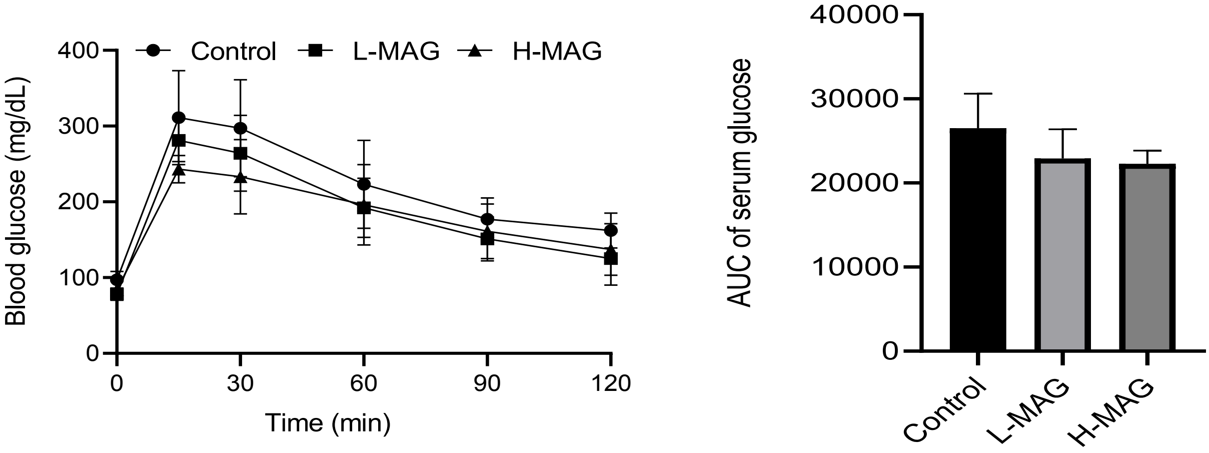

Figure S2. Effects of MAG on blood glucose levels and area under the curve (AUC) of serum glucose from OGTT after a single dose (A) or after 8-week exposure (B) in mice fed with an AIN-93M diet. The OGTT was performed before or after 8-week administration of L-MAG (1.1 g/L) or H-MAG (3.3 g/L) in drinking water, respectively. Values are expressed as mean ± SD (n=4). Differences between groups were determined by one-way ANOVA (p>0.05). OGTT, oral glucose tolerance test; MAG, monoammonium glycyrrhizinate.

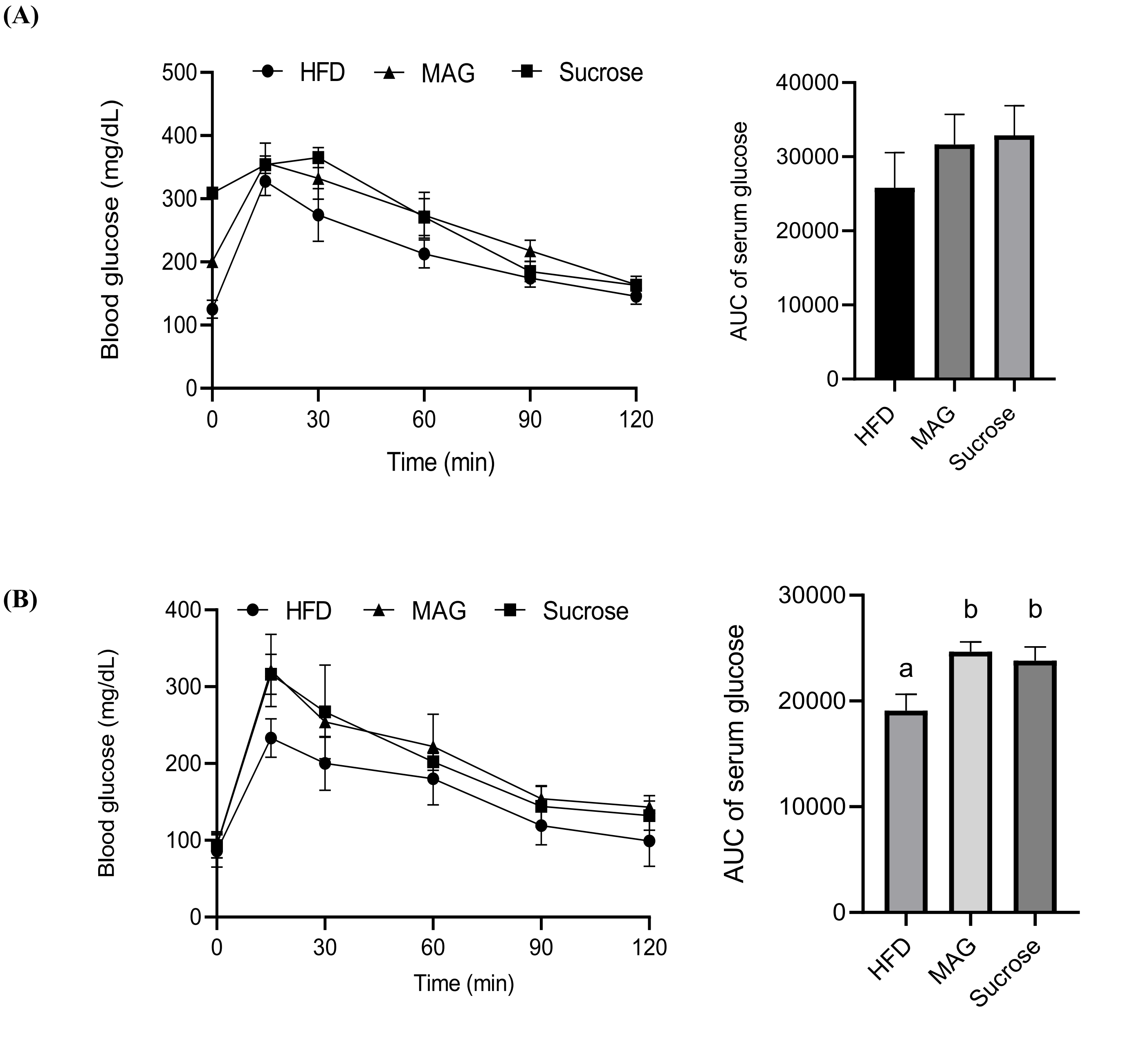

Figure S3. Effects of MAG and sucrose on blood glucose levels and area under the curve (AUC) of serum glucose from OGTT after a single dose (A) or after 8-week exposure (B) in HFD-induced obese mice. After being fed with an HFD for 8 weeks, the animals performed OGTT before or after 8-week administration of MAG (1.1 g/L) or sucrose (266 g/L) in drinking water, respectively. Values are expressed as mean ± SD (n=4). Differences between groups were determined by one-way ANOVA followed by the post hoc Tukey test. ab, Data do not share the same letter significantly differ ($p<0.05$). OGTT, oral glucose tolerance test; MAG, monoammonium glycyrrhizinate; HFD, high-fat diet.
